# Recurrent Unresectable Malignant Granular Cell Tumor With Response to Pazopanib

**DOI:** 10.7759/cureus.8287

**Published:** 2020-05-26

**Authors:** Vatsala Katiyar, Ishaan Vohra, Alok Uprety, Wei Yin, Shweta Gupta

**Affiliations:** 1 Internal Medicine, John H. Stroger, Jr. Hospital of Cook County, Chicago, USA; 2 Internal Medicine, West China School of Medicine, Sichuan University, Sichuan, CHN; 3 Hematology/Oncology, John H. Stroger, Jr. Hospital of Cook County, Chicago, USA

**Keywords:** malignant granular cell tumors, mgct, granular cell tumor, pazopanib, gct

## Abstract

Malignant granular cell tumors (MGCTs) are rare and aggressive variants of granular cell tumors. They usually involve the head and neck region, skin and soft tissues. There are no standard therapeutic guidelines for management; however, surgical resection, whenever feasible, is considered to be first line. We report a patient with recurrent unresectable MGCT of lower lip who responded to pazopanib monotherapy. This drug has been recently approved for the treatment of advanced soft tissue sarcomas. It is a potent oral tyrosine kinase inhibitor and acts on multiple receptors, including vascular endothelial growth factor receptor (VEGFR), epidermal growth factor receptor (EGFR), c-kit, platelet-derived growth factor receptor (PDGFR) and fibroblast growth factor receptor (FGFR). Due to the overexpression of multiple genes by the tumor and multiple targets of this drug, it is difficult to establish the mechanism of action responsible for disease response.

## Introduction

Granular cell tumors (GCTs) are uncommon neoplasms thought to be derived from Schwann cells [1]. Malignant granular cell tumors (MGCTs) are extremely rare (<2% of all GCTs) and are characterized by rapid growth, local destruction and frequent metastasis to regional lymph nodes, lung, liver and bones [2,3]. They are most commonly seen in the head and neck region, skin, subcutaneous and soft tissue [3]. Due to the rarity of disease, there is a lack of randomized controlled trials and standard protocols for treatment. While several chemotherapeutic regimens have been tried, not many have been able to demonstrate sustained clinical response [4]. Hence, the tumor is considered to be relatively chemoresistant, and surgical resection is the treatment of choice [5]. Here we present a patient with recurrent unresectable MGCT who demonstrated radiographic and clinical response to pazopanib.

## Case presentation

A 45-year-old Hispanic man presented to our institution with a progressively enlarging mandibular mass for two years. His medical history was significant for localized MGCT of the lower lip that was diagnosed five years ago in Mexico, for which he underwent surgical resection followed by radiation therapy (RT) and reconstructive surgery. He had a local recurrence one year after initial diagnosis but was lost to follow-up after an excisional biopsy. He presented for medical care two years later to discuss treatment options for the tumor, but it was deemed unresectable. He was treated with palliative chemotherapy (carboplatin, paclitaxel and cetuximab) for unknown number of cycles with no response and was advised hospice. Due to lack of further therapeutic options in Mexico, he presented to us to seek treatment for his advanced disease.

**Figure 1:**
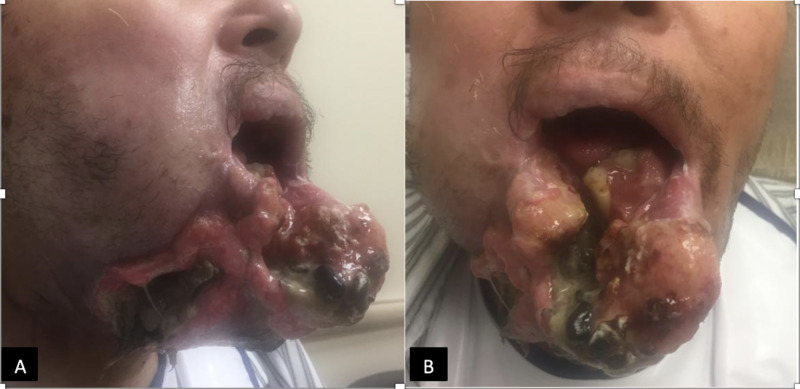
Tumor at presentation to our institute Panes A and B reveal a large fungating mass encompassing the entire lower lip, extending inferiorly to the chin and eroding deep into the floor of mouth.

Physical examination revealed a large, tender exophytic fungating mass approximately 15 cm in size encompassing entire lower lip, extending inferiorly to the chin and inferior angle of mandible on right side and eroding deep into the floor of mouth (Figure 1). Areas of erosions and ulcerations were scattered over the mass expressing serosanguinous discharge. It was associated with decreased tongue mobility, muffled voice and drooling of saliva. However, he was able to swallow soft food and pills. He was not in any respiratory distress. Laboratory workup was unremarkable except for hemoglobin of 10.6 gm/dL (normal 12.9-16.8 gm/dL).

**Figure 2:**
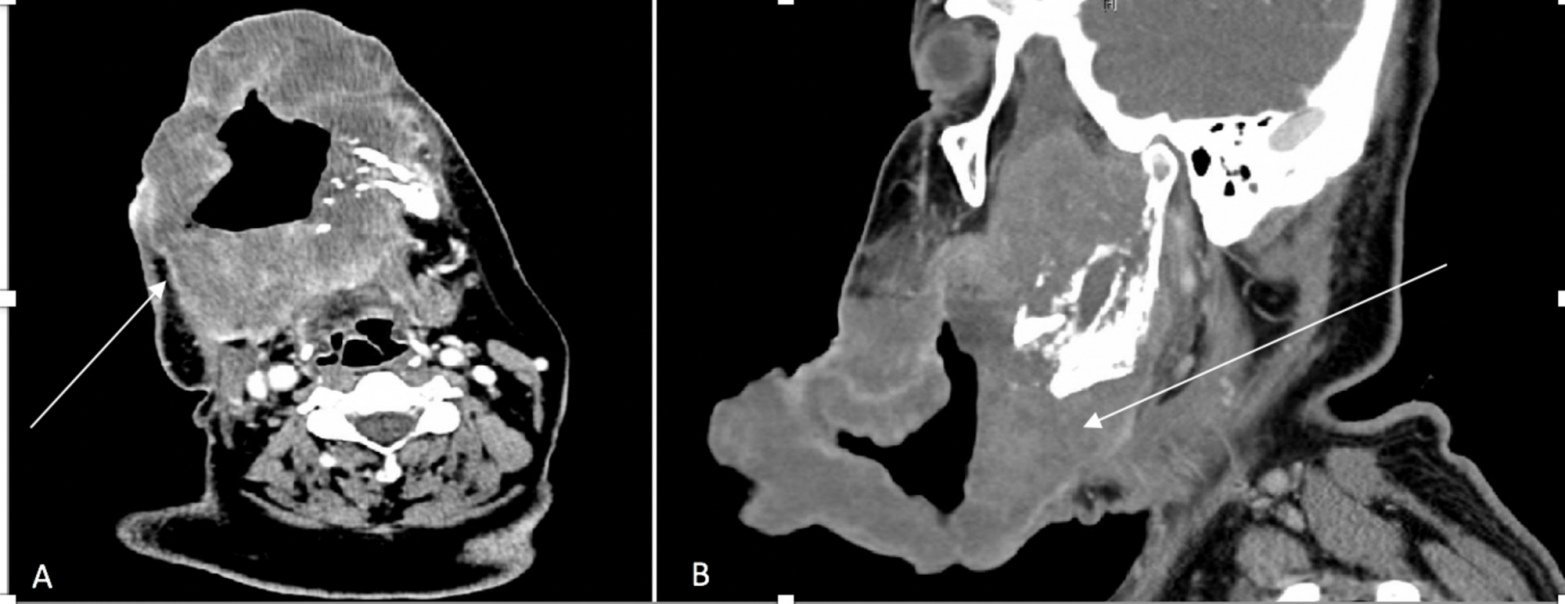
CT scan of neck with intravenous contrast CT scan of neck at presentation: (A) axial and (B) sagittal views. The white arrow points towards large infiltrative heterogeneously enhancing lesion extending from the floor of mouth to the right sided masticator space with associated erosion of the mandible and the right pterygoid plate.

**Figure 3:**
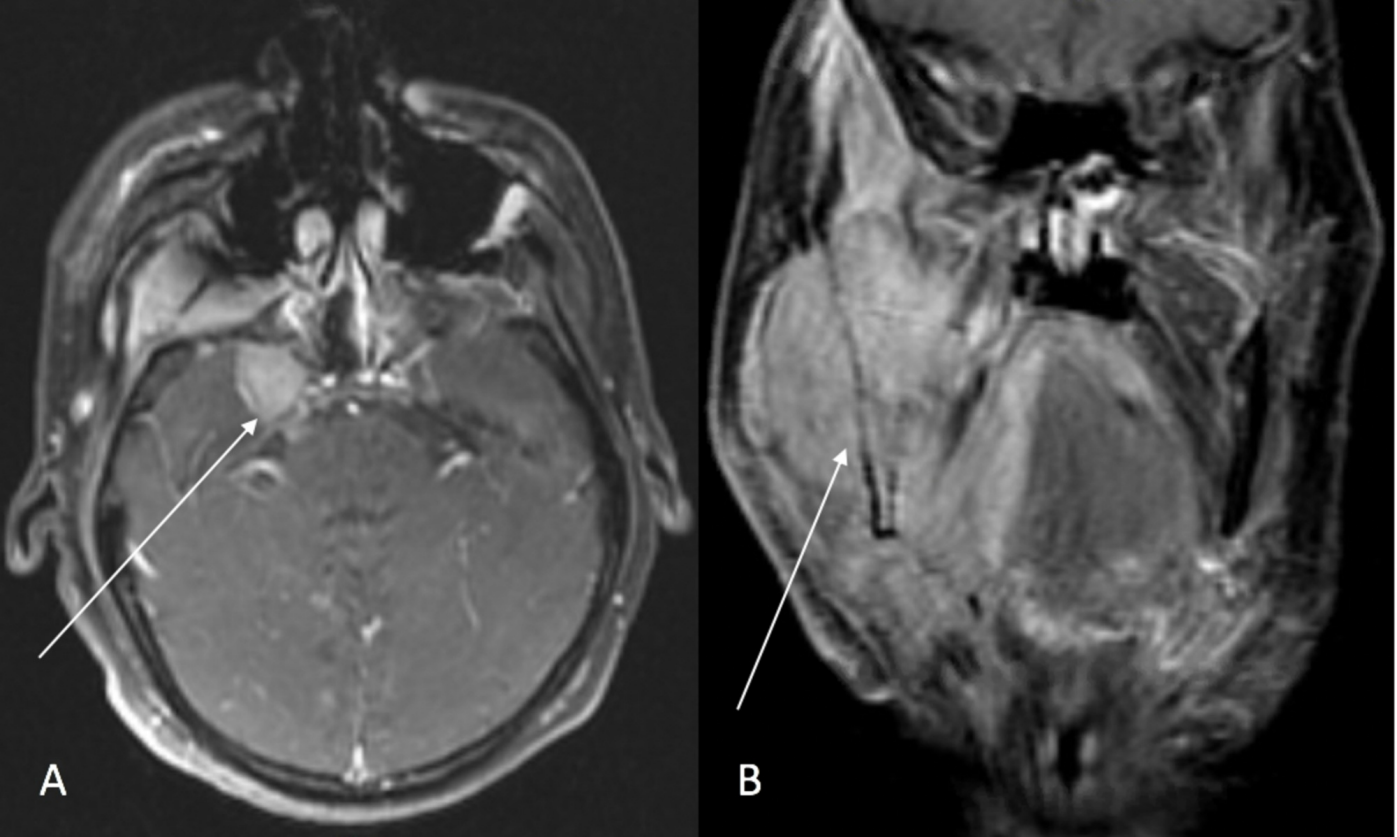
MRI brain and neck with contrast at presentation The white arrow in pane A (axial view) points to the involvement of right Meckel's cave and cisternal segment of the right trigeminal nerve by the tumor. The white arrow in pane B (coronal view) shows the tumor invading into the right masticator space, infrazygomatic space and the oral cavity.

Computed tomography (CT) scan (Figure 2) and magnetic resonance imaging (MRI) of the neck and brain (Figure 3) demonstrated a large infiltrative destructive soft tissue mass involving the right hemimandible, anterior part of left hemimandible, floor of mouth, right oral tongue, masticator space, right temporomandibular joint, skull base, foramen ovale, right Meckel’s cave and cisternal segment of the right trigeminal nerve abutting the pons and bilateral cervical adenopathy. No evidence of metastatic disease was seen on CT scan of chest, abdomen and pelvis. Biopsy of the tumor confirmed MGCT, and it was deemed unresectable. A gastric tube (G-tube) was placed for nutritional support. 

**Figure 4:**
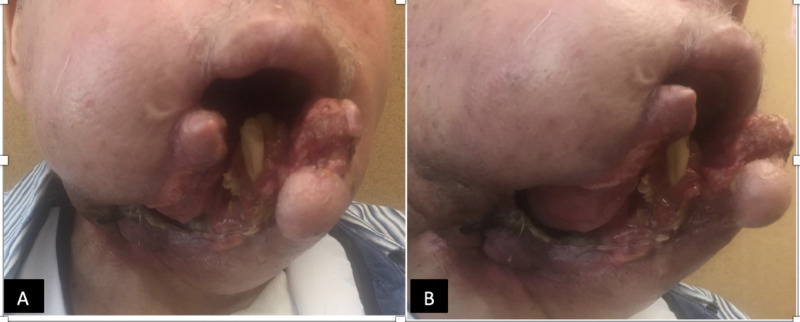
Tumor at six weeks Panes A and B demonstrate the gross appearance of tumor after six weeks of pazopanib initiation. Midline of lower jaw has sloughed off.

The incurable nature of the disease was explained, and he was started on half the usual dose of pazopanib (400 mg) orally due to ongoing bleeding from the tumor, based on the limited available data. It was then increased to 75% of the dose (600 mg) after two weeks. There was a significant improvement in facial swelling, bleeding, discharge and size of the tumor within one month. However, the tumor started to slough off exposing midline part of lower jaw and the tongue resulting in increased pain and trouble swallowing pills (Figure 4). His medications were switched to be taken by G-tube and based on pharmacokinetic data of pazopanib that showed an increase in area under curve by 46% of a crushed tablet, he was prescribed 400 mg (1/2 dose) crushed by G-tube daily. 

**Figure 5:**
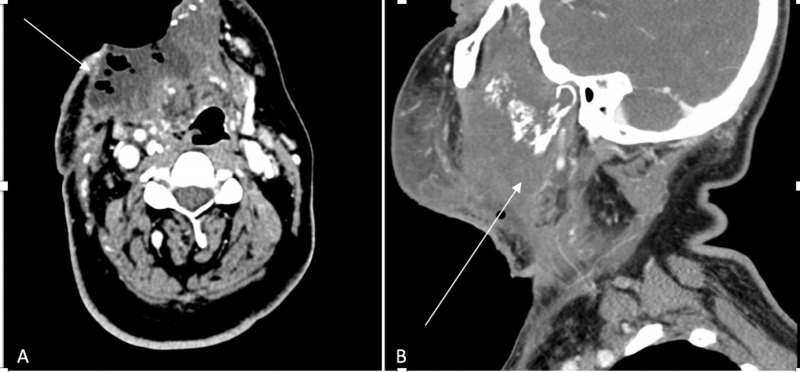
CT scan of neck with contrast after six weeks of therapy CT scan of neck after six weeks of therapy: (A) axial and (B) sagittal views. The white arrow indicates a large necrotic mass involving the patient's right oral cavity, tongue, sublingual space, submandibular space, right masticator space and right pterygopalatine fossa. The mass is more necrotic and has sloughed off.

A CT scan performed after six weeks of treatment initiation revealed a decrease in the size of primary lesion (from 135 x 104 x 127 mm to 105 x 58 x 104 mm) and cervical adenopathy along with improved intracranial extension (Figure 5). Gradually, his tumor stopped sloughing and stabilized. Secretions and facial swelling continued to diminish and pain was well controlled with narcotics. Repeat CT scans done at three months after the initiation of pazopanib showed overall radiological improvement of local disease and a slight decrease in intracranial extension.

**Figure 6:**
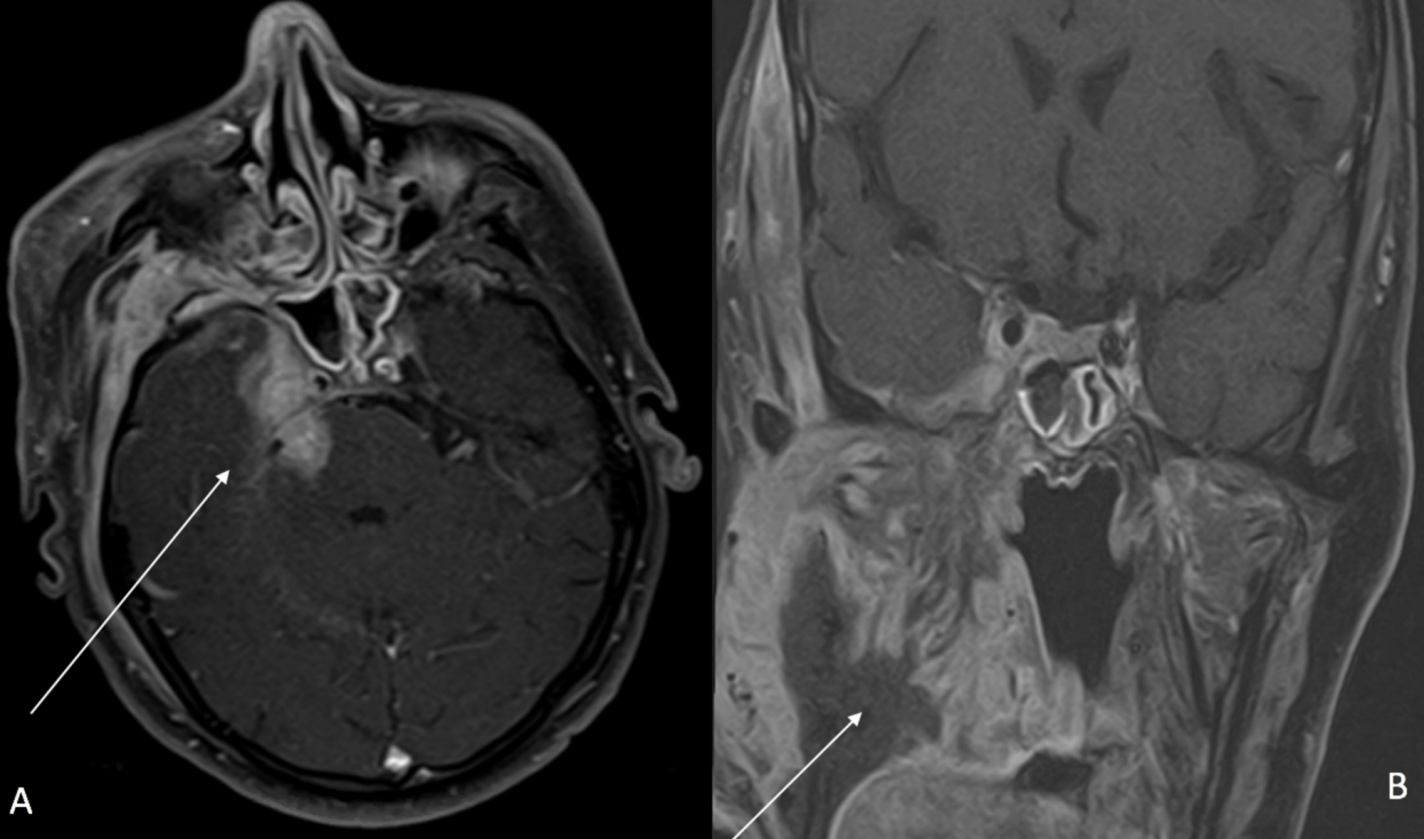
MRI brain and neck with contrast four months after pazopanib therapy The arrow in pane A (axial view) highlights the increased intracranial burden of disease with involvement of right Meckel's cave, right cavernous sinus and frank invasion into the ventral pons. The arrow in pane B (coronal view) points to the mild decrease in tumor burden extracranially especially in the right infrazygomatic masticator space and tumor necrosis.

After four months of treatment, he developed diplopia and right lateral rectus palsy. MRI brain and neck revealed interval increase in intracranial disease, invasion of right cavernous sinus, pons and abducens nerve, while redemonstrating a decrease in extracranial tumor burden, especially in the infrazygomatic masticator space (Figure 6). He was treated with dexamethasone, 9 Gy of RT and tarsorrhaphy. Pazopanib was increased to 600 mg daily. At nine months, his CT scan showed stable intracranial disease, while his extracranial disease became more necrotic. He did not report any adverse events from pazopanib. He passed away suddenly at home 10 months after the initiation of pazopanib. The family refused an autopsy. 

## Discussion

MGCTs are extremely rare and aggressive variants of GCTs with a predilection for recurrence and metastasis [2]. They are seen more often in females and typically involve the head and neck region, skin and soft tissues [3]. While metastatic GCTs are considered malignant, histological criteria for diagnosis of MGCT exists as well. Fanburg-Smith proposed the following histologic characteristics to assess the malignant potential: increased mitotic activity, necrosis, tumor cell spindling, vesicular nuclei with prominent nucleoli, increased nucleus to cytoplasm ratio and pleomorphism. The presence of three or more criteria indicates malignancy [2]. These tumors tend to have unfavorable prognosis. Fanburg-Smith et al reported that 39% of patients died at three years, 50% experienced metastases and 32% developed local recurrence [2]. Regional lymph nodes, lung, liver and bones are regarded as the most common sites of metastases [3].

MGCTs are relatively chemoinsensitive, and surgical excision is considered to be the initial treatment of choice for localized tumors [5]. Standard therapy protocols do not exist due to rarity of disease and lack of randomized control trials. There is very limited data to support the role of systemic therapies. Our patient failed RT and palliative chemotherapy. We used pazopanib based on few case reports that demonstrated clinical and radiographic response in patients treated with this tyrosine kinase inhibitor. To the best of our knowledge, there have been three previously reported cases of a positive response to pazopanib in MGCT [5-7].

Pazopanib is an oral agent that targets receptors for vascular endothelial growth factor, platelet-derived growth factor and c-kit amongst others. The European Organisation for Research and Treatment of Cancer (EORTC) study 62043 was a phase II clinical trial, which showed that it has activity against relapsed and refractory soft tissue sarcomas [8]. Subsequently, a phase III trial, pazopanib for metastatic soft-tissue sarcoma (PALETTE), showed that pazopanib could be used for treatment of soft tissue sarcomas [9]. It has demonstrated response in patients with MGCT, but cure has not been documented [5-7]. Our patient too exhibited clinical and radiological improvement. Notably, while the extracranial tumor burden decreased, his intracranial disease progressed leading to involvement of abducens nerve, finally stabilizing after RT. The progression was likely due to the inability of pazopanib to cross the blood-brain barrier. However, we are unable to explain the initial improvement in the intracranial disease. In addition, the pazopanib dose studied for soft tissue sarcomas in the PALETTE trial is 800 mg; however, our patient showed initial clinical response with 50% of the dose and subsequently 75% of the dose possibly suggesting high sensitivity of this tumor to pazopanib [9].

Unfortunately, only limited genetic information regarding the molecular nature of the tumor is available. Whole-genome sequencing of an MGCT has exhibited an overall stable genome with no identifiable mutations in the molecular targets of pazopanib. In the same tumor, a loss of function of bromodomain-containing protein 7 (BRD7) gene and tyrosine kinase pathway mutation in GFRA2 was noted, which needs further investigation [7]. Genetic assays were not performed in our patient. Since pazopanib is a multikinase inhibitor and overexpression of multiple genes can be seen in the malignancy, it is difficult to ascertain the true mechanism responsible for tumor response.

## Conclusions

Rarity of MGCT makes it difficult to have set standard treatment protocols. It is, however, a relatively chemoresistant tumor with curative resection being the cornerstone for therapy. Better characterization of the genetics and driver mutations can help tailor and personalize therapy for patients. Further studies are required to clearly identify the benefits of pazopanib and other chemotherapy regimens in MGCT as well as to establish the best form of multimodality treatment. 
